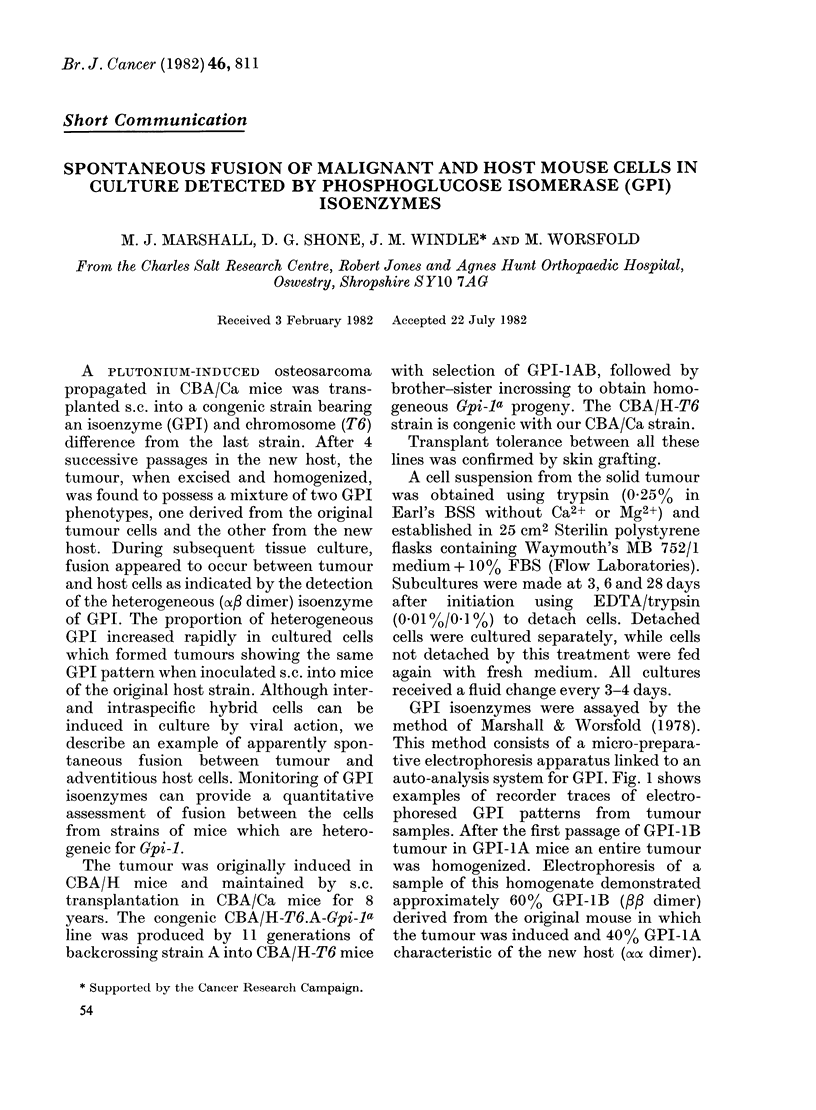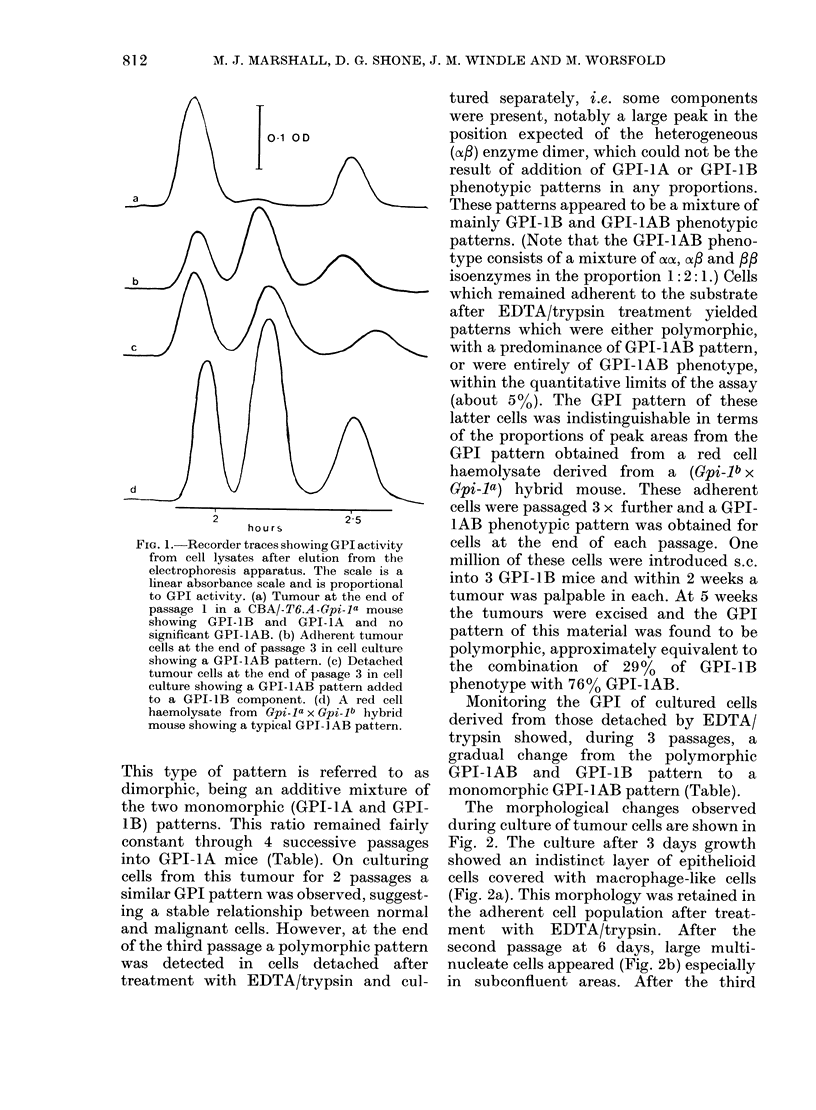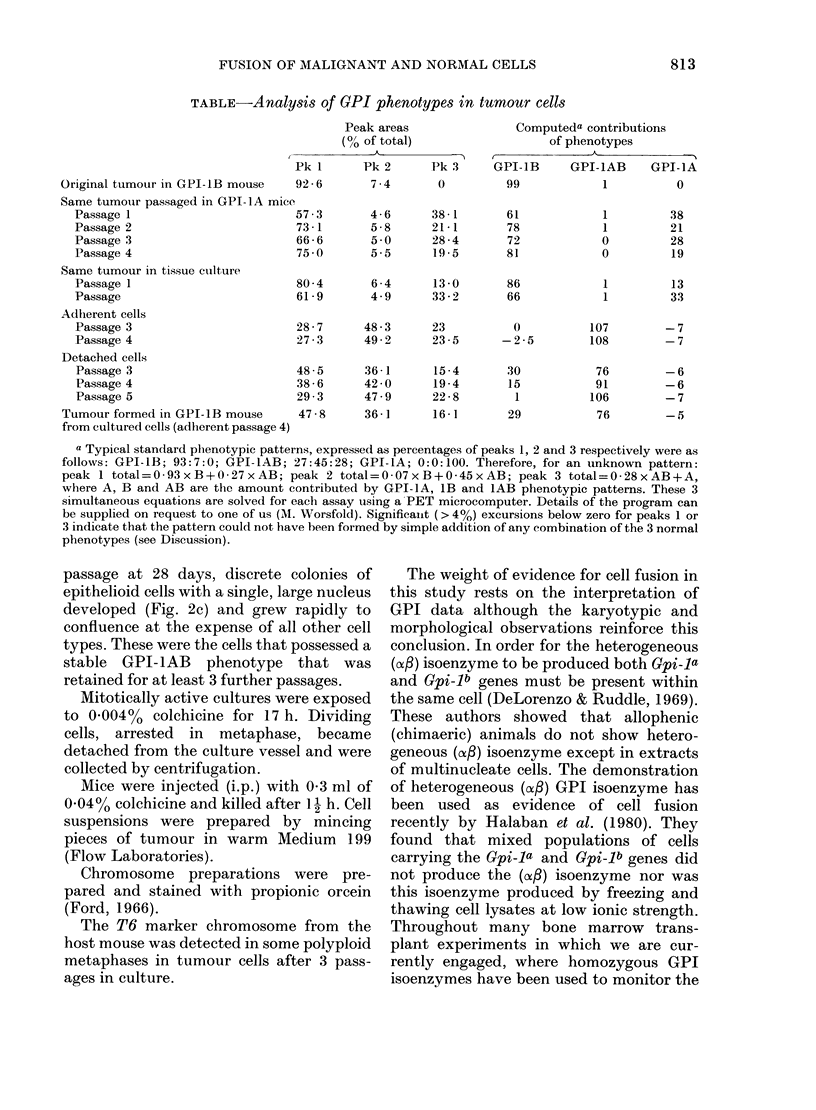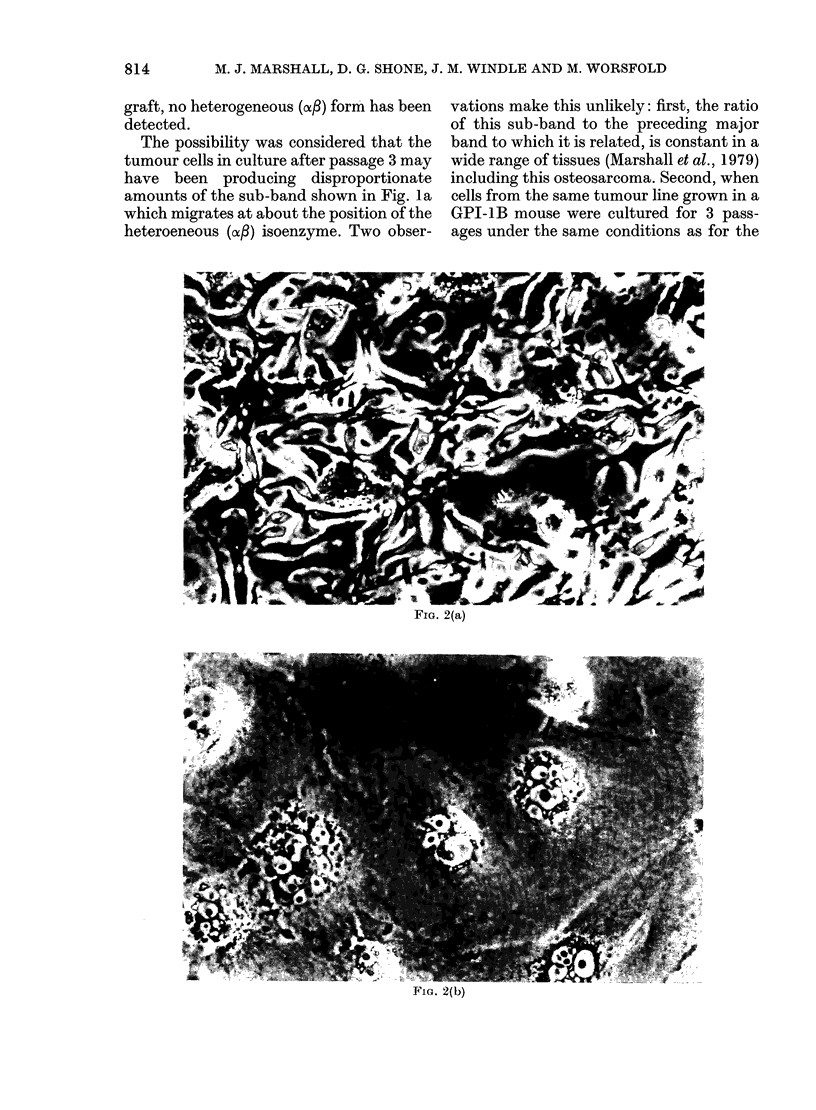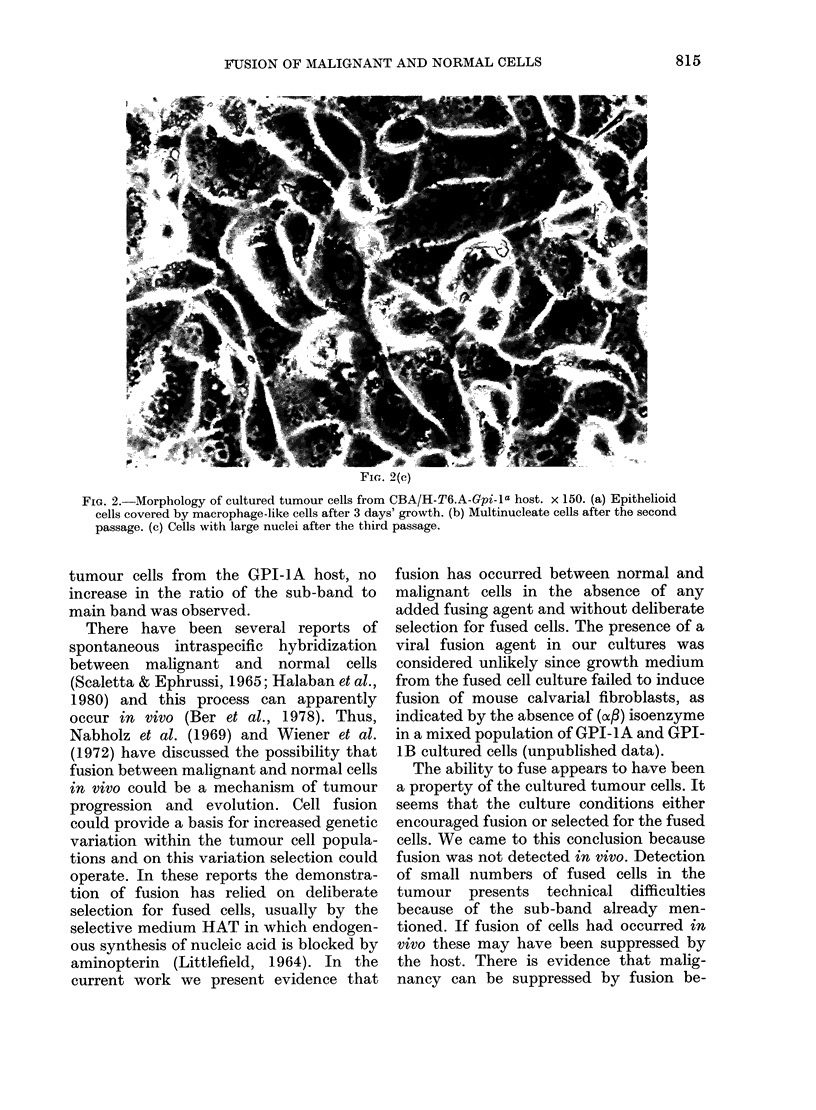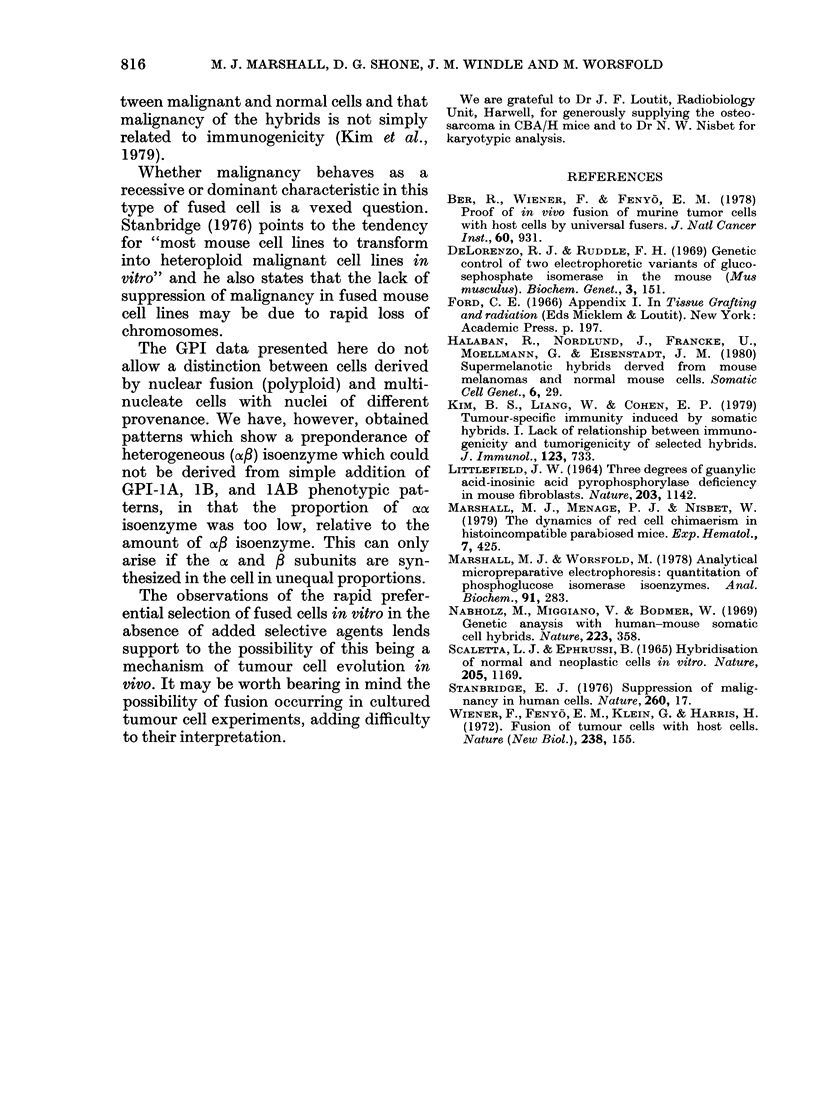# Spontaneous fusion of malignant and host mouse cells in culture detected by phosphoglucose isomerase (GPI) isoenzymes.

**DOI:** 10.1038/bjc.1982.275

**Published:** 1982-11

**Authors:** M. J. Marshall, D. G. Shone, J. M. Windle, M. Worsfold

## Abstract

**Images:**


*Br. J. Cancer* (1982) **46**, 811

### *Short Communication*

### SPONTANEOUS FUSION OF MALIGNANT AND HOST MOUSE CELLS IN CULTURE DETECTED BY PHOSPHOGLUCOSE ISOMERASE (GPI) ISOENZYMES

M. J. MARSHALL, D. G. SHONE, J. M. WINDLE* AND M. WORSFOLD

*From the Charles Salt Research Centre, Robert Jones and Agnes Hunt Orthopaedic Hospital, Oswestry, Shropshire SY10 7AG*

Received 3 February 1982  Accepted 22 July 1982

A PLUTONIUM-INDUCED osteosarcoma propagated in CBA/Ca mice was transplanted s.c. into a congenic strain bearing an isoenzyme (GPI) and chromosome (*T6*) difference from the last strain. After 4 successive passages in the new host, the tumour, when excised and homogenized, was found to possess a mixture of two GPI phenotypes, one derived from the original tumour cells and the other from the new host. During subsequent tissue culture, fusion appeared to occur between tumour and host cells as indicated by the detection of the heterogeneous ($\alpha\beta$ dimer) isoenzyme of GPI. The proportion of heterogeneous GPI increased rapidly in cultured cells which formed tumours showing the same GPI pattern when inoculated s.c. into mice of the original host strain. Although inter- and intraspecific hybrid cells can be induced in culture by viral action, we describe an example of apparently spontaneous fusion between tumour and adventitious host cells. Monitoring of GPI isoenzymes can provide a quantitative assessment of fusion between the cells from strains of mice which are heterogeneic for *Gpi-1*.

The tumour was originally induced in CBA/H mice and maintained by s.c. transplantation in CBA/Ca mice for 8 years. The congenic CBA/H-*T6*.A-*Gpi-1$^a$* line was produced by 11 generations of backcrossing strain A into CBA/H-*T6* mice with selection of GPI-1AB, followed by brother–sister incrossing to obtain homogeneous *Gpi-1$^a$* progeny. The CBA/H-*T6* strain is congenic with our CBA/Ca strain.

Transplant tolerance between all these lines was confirmed by skin grafting.

A cell suspension from the solid tumour was obtained using trypsin (0·25% in Earl's BSS without $Ca^{2+}$ or $Mg^{2+}$) and established in 25 $cm^2$ Sterilin polystyrene flasks containing Waymouth's MB 752/1 medium + 10% FBS (Flow Laboratories). Subcultures were made at 3, 6 and 28 days after initiation using EDTA/trypsin (0·01%/0·1%) to detach cells. Detached cells were cultured separately, while cells not detached by this treatment were fed again with fresh medium. All cultures received a fluid change every 3–4 days.

GPI isoenzymes were assayed by the method of Marshall & Worsfold (1978). This method consists of a micro-preparative electrophoresis apparatus linked to an auto-analysis system for GPI. Fig. 1 shows examples of recorder traces of electrophoresed GPI patterns from tumour samples. After the first passage of GPI-1B tumour in GPI-1A mice an entire tumour was homogenized. Electrophoresis of a sample of this homogenate demonstrated approximately 60% GPI-1B ($\beta\beta$ dimer) derived from the original mouse in which the tumour was induced and 40% GPI-1A characteristic of the new host ($\alpha\alpha$ dimer).

* Supported by the Cancer Research Campaign.

FIG. 1.—Recorder traces showing GPI activity from cell lysates after elution from the electrophoresis apparatus. The scale is a linear absorbance scale and is proportional to GPI activity. (a) Tumour at the end of passage 1 in a CBA/-*T6.A-Gpi-1*$^{a}$ mouse showing GPI-1B and GPI-1A and no significant GPI-1AB. (b) Adherent tumour cells at the end of passage 3 in cell culture showing a GPI-1AB pattern. (c) Detached tumour cells at the end of pasage 3 in cell culture showing a GPI-1AB pattern added to a GPI-1B component. (d) A red cell haemolysate from *Gpi-1*$^{a}$ × *Gpi-1*$^{b}$ hybrid mouse showing a typical GPI-1AB pattern.

This type of pattern is referred to as dimorphic, being an additive mixture of the two monomorphic (GPI-1A and GPI-1B) patterns. This ratio remained fairly constant through 4 successive passages into GPI-1A mice (Table). On culturing cells from this tumour for 2 passages a similar GPI pattern was observed, suggesting a stable relationship between normal and malignant cells. However, at the end of the third passage a polymorphic pattern was detected in cells detached after treatment with EDTA/trypsin and cultured separately, *i.e.* some components were present, notably a large peak in the position expected of the heterogeneous ($\alpha\beta$) enzyme dimer, which could not be the result of addition of GPI-1A or GPI-1B phenotypic patterns in any proportions. These patterns appeared to be a mixture of mainly GPI-1B and GPI-1AB phenotypic patterns. (Note that the GPI-1AB phenotype consists of a mixture of $\alpha\alpha$, $\alpha\beta$ and $\beta\beta$ isoenzymes in the proportion 1:2:1.) Cells which remained adherent to the substrate after EDTA/trypsin treatment yielded patterns which were either polymorphic, with a predominance of GPI-1AB pattern, or were entirely of GPI-1AB phenotype, within the quantitative limits of the assay (about 5%). The GPI pattern of these latter cells was indistinguishable in terms of the proportions of peak areas from the GPI pattern obtained from a red cell haemolysate derived from a (*Gpi-1*$^{b}$ × *Gpi-1*$^{a}$) hybrid mouse. These adherent cells were passaged 3 × further and a GPI-1AB phenotypic pattern was obtained for cells at the end of each passage. One million of these cells were introduced s.c. into 3 GPI-1B mice and within 2 weeks a tumour was palpable in each. At 5 weeks the tumours were excised and the GPI pattern of this material was found to be polymorphic, approximately equivalent to the combination of 29% of GPI-1B phenotype with 76% GPI-1AB.

Monitoring the GPI of cultured cells derived from those detached by EDTA/trypsin showed, during 3 passages, a gradual change from the polymorphic GPI-1AB and GPI-1B pattern to a monomorphic GPI-1AB pattern (Table).

The morphological changes observed during culture of tumour cells are shown in Fig. 2. The culture after 3 days growth showed an indistinct layer of epithelioid cells covered with macrophage-like cells (Fig. 2a). This morphology was retained in the adherent cell population after treatment with EDTA/trypsin. After the second passage at 6 days, large multinucleate cells appeared (Fig. 2b) especially in subconfluent areas. After the third

TABLE—*Analysis of GPI phenotypes in tumour cells*

| | Peak areas (% of total) | | | Computed[a] contributions of phenotypes | | |
|---|---|---|---|---|---|---|
| | Pk 1 | Pk 2 | Pk 3 | GPI-1B | GPI-1AB | GPI-1A |
| Original tumour in GPI-1B mouse | 92·6 | 7·4 | 0 | 99 | 1 | 0 |
| Same tumour passaged in GPI-1A mice | | | | | | |
| Passage 1 | 57·3 | 4·6 | 38·1 | 61 | 1 | 38 |
| Passage 2 | 73·1 | 5·8 | 21·1 | 78 | 1 | 21 |
| Passage 3 | 66·6 | 5·0 | 28·4 | 72 | 0 | 28 |
| Passage 4 | 75·0 | 5·5 | 19·5 | 81 | 0 | 19 |
| Same tumour in tissue culture | | | | | | |
| Passage 1 | 80·4 | 6·4 | 13·0 | 86 | 1 | 13 |
| Passage | 61·9 | 4·9 | 33·2 | 66 | 1 | 33 |
| Adherent cells | | | | | | |
| Passage 3 | 28·7 | 48·3 | 23 | 0 | 107 | −7 |
| Passage 4 | 27·3 | 49·2 | 23·5 | −2·5 | 108 | −7 |
| Detached cells | | | | | | |
| Passage 3 | 48·5 | 36·1 | 15·4 | 30 | 76 | −6 |
| Passage 4 | 38·6 | 42·0 | 19·4 | 15 | 91 | −6 |
| Passage 5 | 29·3 | 47·9 | 22·8 | 1 | 106 | −7 |
| Tumour formed in GPI-1B mouse from cultured cells (adherent passage 4) | 47·8 | 36·1 | 16·1 | 29 | 76 | −5 |

[a] Typical standard phenotypic patterns, expressed as percentages of peaks 1, 2 and 3 respectively were as follows: GPI-1B; 93:7:0; GPI-1AB; 27:45:28; GPI-1A; 0:0:100. Therefore, for an unknown pattern: peak 1 total $= 0{\cdot}93 \times B + 0{\cdot}27 \times AB$; peak 2 total $= 0{\cdot}07 \times B + 0{\cdot}45 \times AB$; peak 3 total $= 0{\cdot}28 \times AB + A$, where A, B and AB are the amount contributed by GPI-1A, 1B and 1AB phenotypic patterns. These 3 simultaneous equations are solved for each assay using a PET microcomputer. Details of the program can be supplied on request to one of us (M. Worsfold). Significant ($>4\%$) excursions below zero for peaks 1 or 3 indicate that the pattern could not have been formed by simple addition of any combination of the 3 normal phenotypes (see Discussion).

passage at 28 days, discrete colonies of epithelioid cells with a single, large nucleus developed (Fig. 2c) and grew rapidly to confluence at the expense of all other cell types. These were the cells that possessed a stable GPI-1AB phenotype that was retained for at least 3 further passages.

Mitotically active cultures were exposed to 0·004% colchicine for 17 h. Dividing cells, arrested in metaphase, became detached from the culture vessel and were collected by centrifugation.

Mice were injected (i.p.) with 0·3 ml of 0·04% colchicine and killed after $1\frac{1}{2}$ h. Cell suspensions were prepared by mincing pieces of tumour in warm Medium 199 (Flow Laboratories).

Chromosome preparations were prepared and stained with propionic orcein (Ford, 1966).

The *T6* marker chromosome from the host mouse was detected in some polyploid metaphases in tumour cells after 3 passages in culture.

The weight of evidence for cell fusion in this study rests on the interpretation of GPI data although the karyotypic and morphological observations reinforce this conclusion. In order for the heterogeneous ($\alpha\beta$) isoenzyme to be produced both *Gpi-1*$^{a}$ and *Gpi-1*$^{b}$ genes must be present within the same cell (DeLorenzo & Ruddle, 1969). These authors showed that allophenic (chimaeric) animals do not show heterogeneous ($\alpha\beta$) isoenzyme except in extracts of multinucleate cells. The demonstration of heterogeneous ($\alpha\beta$) GPI isoenzyme has been used as evidence of cell fusion recently by Halaban *et al.* (1980). They found that mixed populations of cells carrying the *Gpi-1*$^{a}$ and *Gpi-1*$^{b}$ genes did not produce the ($\alpha\beta$) isoenzyme nor was this isoenzyme produced by freezing and thawing cell lysates at low ionic strength. Throughout many bone marrow transplant experiments in which we are currently engaged, where homozygous GPI isoenzymes have been used to monitor the graft, no heterogeneous ($\alpha\beta$) form has been detected.

The possibility was considered that the tumour cells in culture after passage 3 may have been producing disproportionate amounts of the sub-band shown in Fig. 1a which migrates at about the position of the heteroeneous ($\alpha\beta$) isoenzyme. Two observations make this unlikely: first, the ratio of this sub-band to the preceding major band to which it is related, is constant in a wide range of tissues (Marshall *et al.*, 1979) including this osteosarcoma. Second, when cells from the same tumour line grown in a GPI-1B mouse were cultured for 3 passages under the same conditions as for the

FIG. 2(a)

FIG. 2(b)

FIG. 2(c)

FIG. 2.—Morphology of cultured tumour cells from CBA/H-*T*6.A-*Gpi*-$1^a$ host. ×150. (a) Epithelioid cells covered by macrophage-like cells after 3 days' growth. (b) Multinucleate cells after the second passage. (c) Cells with large nuclei after the third passage.

tumour cells from the GPI-1A host, no increase in the ratio of the sub-band to main band was observed.

There have been several reports of spontaneous intraspecific hybridization between malignant and normal cells (Scaletta & Ephrussi, 1965; Halaban *et al.*, 1980) and this process can apparently occur *in vivo* (Ber *et al.*, 1978). Thus, Nabholz *et al.* (1969) and Wiener *et al.* (1972) have discussed the possibility that fusion between malignant and normal cells *in vivo* could be a mechanism of tumour progression and evolution. Cell fusion could provide a basis for increased genetic variation within the tumour cell populations and on this variation selection could operate. In these reports the demonstration of fusion has relied on deliberate selection for fused cells, usually by the selective medium HAT in which endogenous synthesis of nucleic acid is blocked by aminopterin (Littlefield, 1964). In the current work we present evidence that fusion has occurred between normal and malignant cells in the absence of any added fusing agent and without deliberate selection for fused cells. The presence of a viral fusion agent in our cultures was considered unlikely since growth medium from the fused cell culture failed to induce fusion of mouse calvarial fibroblasts, as indicated by the absence of ($\alpha\beta$) isoenzyme in a mixed population of GPI-1A and GPI-1B cultured cells (unpublished data).

The ability to fuse appears to have been a property of the cultured tumour cells. It seems that the culture conditions either encouraged fusion or selected for the fused cells. We came to this conclusion because fusion was not detected *in vivo*. Detection of small numbers of fused cells in the tumour presents technical difficulties because of the sub-band already mentioned. If fusion of cells had occurred *in vivo* these may have been suppressed by the host. There is evidence that malignancy can be suppressed by fusion between malignant and normal cells and that malignancy of the hybrids is not simply related to immunogenicity (Kim *et al.*, 1979).

Whether malignancy behaves as a recessive or dominant characteristic in this type of fused cell is a vexed question. Stanbridge (1976) points to the tendency for "most mouse cell lines to transform into heteroploid malignant cell lines *in vitro*" and he also states that the lack of suppression of malignancy in fused mouse cell lines may be due to rapid loss of chromosomes.

The GPI data presented here do not allow a distinction between cells derived by nuclear fusion (polyploid) and multinucleate cells with nuclei of different provenance. We have, however, obtained patterns which show a preponderance of heterogeneous ($\alpha\beta$) isoenzyme which could not be derived from simple addition of GPI-1A, 1B, and 1AB phenotypic patterns, in that the proportion of $\alpha\alpha$ isoenzyme was too low, relative to the amount of $\alpha\beta$ isoenzyme. This can only arise if the $\alpha$ and $\beta$ subunits are synthesized in the cell in unequal proportions.

The observations of the rapid preferential selection of fused cells *in vitro* in the absence of added selective agents lends support to the possibility of this being a mechanism of tumour cell evolution *in vivo*. It may be worth bearing in mind the possibility of fusion occurring in cultured tumour cell experiments, adding difficulty to their interpretation.

We are grateful to Dr J. F. Loutit, Radiobiology Unit, Harwell, for generously supplying the osteosarcoma in CBA/H mice and to Dr N. W. Nisbet for karyotypic analysis.

### REFERENCES

BER, R., WIENER, F. & FENYÖ, E. M. (1978) Proof of *in vivo* fusion of murine tumor cells with host cells by universal fusers. *J. Natl Cancer Inst.*, **60**, 931.

DELORENZO, R. J. & RUDDLE, F. H. (1969) Genetic control of two electrophoretic variants of glucosephosphate isomerase in the mouse (*Mus musculus*). *Biochem. Genet.*, **3**, 151.

FORD, C. E. (1966) Appendix I. In *Tissue Grafting and radiation* (Eds Micklem & Loutit). New York: Academic Press. p. 197.

HALABAN, R., NORDLUND, J., FRANCKE, U., MOELLMANN, G. & EISENSTADT, J. M. (1980) Supermelanotic hybrids derved from mouse melanomas and normal mouse cells. *Somatic Cell Genet.*, **6**, 29.

KIM, B. S., LIANG, W. & COHEN, E. P. (1979) Tumour-specific immunity induced by somatic hybrids. I. Lack of relationship between immunogenicity and tumorigenicity of selected hybrids. *J. Immunol.*, **123**, 733.

LITTLEFIELD, J. W. (1964) Three degrees of guanylic acid-inosinic acid pyrophosphorylase deficiency in mouse fibroblasts. *Nature*, **203**, 1142.

MARSHALL, M. J., MENAGE, P. J. & NISBET, W. (1979) The dynamics of red cell chimaerism in histoincompatible parabiosed mice. *Exp. Hematol.*, **7**, 425.

MARSHALL, M. J. & WORSFOLD, M. (1978) Analytical micropreparative electrophoresis: quantitation of phosphoglucose isomerase isoenzymes. *Anal. Biochem.*, **91**, 283.

NABHOLZ, M., MIGGIANO, V. & BODMER, W. (1969) Genetic anaysis with human–mouse somatic cell hybrids. *Nature*, **223**, 358.

SCALETTA, L. J. & EPHRUSSI, B. (1965) Hybridisation of normal and neoplastic cells *in vitro*. *Nature*, **205**, 1169.

STANBRIDGE, E. J. (1976) Suppression of malignancy in human cells. *Nature*, **260**, 17.

WIENER, F., FENYÖ, E. M., KLEIN, G. & HARRIS, H. (1972). Fusion of tumour cells with host cells. *Nature* (*New Biol.*), **238**, 155.